# Spatiotemporal control of gene expression in bone-marrow derived cells of the tumor microenvironment induced by MRI guided focused ultrasound

**DOI:** 10.18632/oncotarget.4288

**Published:** 2015-06-15

**Authors:** Pierre-Yves Fortin, Matthieu Lepetit-Coiffé, Coralie Genevois, Christelle Debeissat, Bruno Quesson, Chrit T.W. Moonen, Jan Pieter Konsman, Franck Couillaud

**Affiliations:** ^1^ Laboratoire d'Imagerie Moléculaire et Fonctionnelle (IMF), CNRS/UMR 5231, Université de Bordeaux, Bordeaux, France; ^2^ Institut de Bio-Imagerie (IBIO), CNRS/UMS 3428, Université de Bordeaux, Bordeaux, France; ^3^ Centre de Résonance Magnétique des Systèmes Biologiques (RMSB), CNRS/UMR 5536, Université de Bordeaux, Bordeaux, France

**Keywords:** molecular imaging, gene therapy, cancer

## Abstract

The tumor microenvironment is an interesting target for anticancer therapies but modifying this compartment is challenging. Here, we demonstrate the feasibility of a gene therapy strategy that combined targeting to bone marrow-derived tumor microenvironment using genetically modified bone-marrow derived cells and control of transgene expression by local hyperthermia through a thermo-inducible promoter. Chimera were obtained by engraftment of bone marrow from transgenic mice expressing reporter genes under transcriptional control of heat shock promoter and inoculated sub-cutaneously with tumors cells. Heat shocks were applied at the tumor site using a water bath or magnetic resonance guided high intensity focused ultrasound device. Reporter gene expression was followed by bioluminescence and fluorescence imaging and immunohistochemistry. Bone marrow-derived cells expressing reporter genes were identified to be mainly tumor-associated macrophages. We thus provide the proof of concept for a gene therapy strategy that allows for spatiotemporal control of transgenes expression by macrophages targeted to the tumor microenvironment.

## INTRODUCTION

Cancer development is determined not only by the tumor cell genotype but also by interactions with tumor microenvironment or stroma [1–3]. The tumor microenvironment initially exhibits a growth-suppressive state but stromal cells may later become educated by the tumor to acquire pro-tumorigenic properties [1, 3]. However, the microenvironment is also able to normalize tumor cells, suggesting that re-education of stromal cells may be an effective strategy for treating cancer [3].

Tumor-associated macrophages (TAMs) are migratory hematopoietic cells originating from bone marrow myeloid precursors and constitute a large part of the tumor microenvironment. TAMs are first recruited from circulating monocytes to reject the tumor after it has been recognized [4–6] but later on, they play a complex and crucial role in tumor progression, metastasis and inflammation [7]. The phenotype of TAMs varies with the stage of tumor development, with M1-like cells often predominating at sites of chronic inflammation where tumors can develop, then switching to an M2-like phenotype as the tumor begins to invade, vascularize and develop [8, 9]. Macrophages therefore represent both an important drug target and a potential cell vector for cancer treatment and prevention of metastases. Genetic modification of hematopoietic stem cells, myeloid precursors or TAMs themselves to express potential anticancer transgenes may be used to modify tumor microenvironment and, thus to influence tumor fate. However, the complex physio-pathological role of macrophages, their broad localization in both healthy and diseased tissues and their versatile phenotype require strategies to restrict transgene expression to the tumor location and in time.

Hyperthermia coupled with a thermo-sensitive heat shock (HSP) promoter offers an unique approach to perform non-invasive spatio-temporal control of transgene activation [10, 11]. High Intensity Focused Ultrasound guided by Magnetic Resonance thermometry (MRgHIFU) allows for accurate and precise, non-invasive *in vivo* local heating, and thus for local activation of gene expression [12, 13].

The aims of this study were to show using *in vivo* monitoring by optical imaging of a chimera mouse model 1) that genetically modified bone marrow cells are enriched in solid tumor micro environment and 2) that hyperthermia allows for spatially and temporally restricted gene expression by TAMs in tumor microenvironment.

## RESULTS

### Chimera mouse generation and tumor growth

Chimera mice, chemo-depleted and engrafted with bone marrow from hsp-lucF (*n* = 49) or hsp-lucF/hsp-mPlum mice (*n* = 18) were assessed 1 month after engraftment by flow cytometry to determine the CD45.2 (donor) CD45.1 (recipient) ratio in circulating blood cells. Mice exhibiting engraftment efficiency higher than 65% (*n* = 35 and *n* = 13 for bone marrow from hsp-lucF and hsp-lucF/hsp-mPlum mice respectively) were chosen for subcutaneous implantation of 2.10^6^ CMT-93 cells on the left leg. Tumor growth occurred in 100% of injected mice and time required to reach a tumor diameter of about 10 mm is 30 +/− 10 days.

### Reporter gene expression after heating the tumor-bearing leg in a water bath

**Figure 1:**
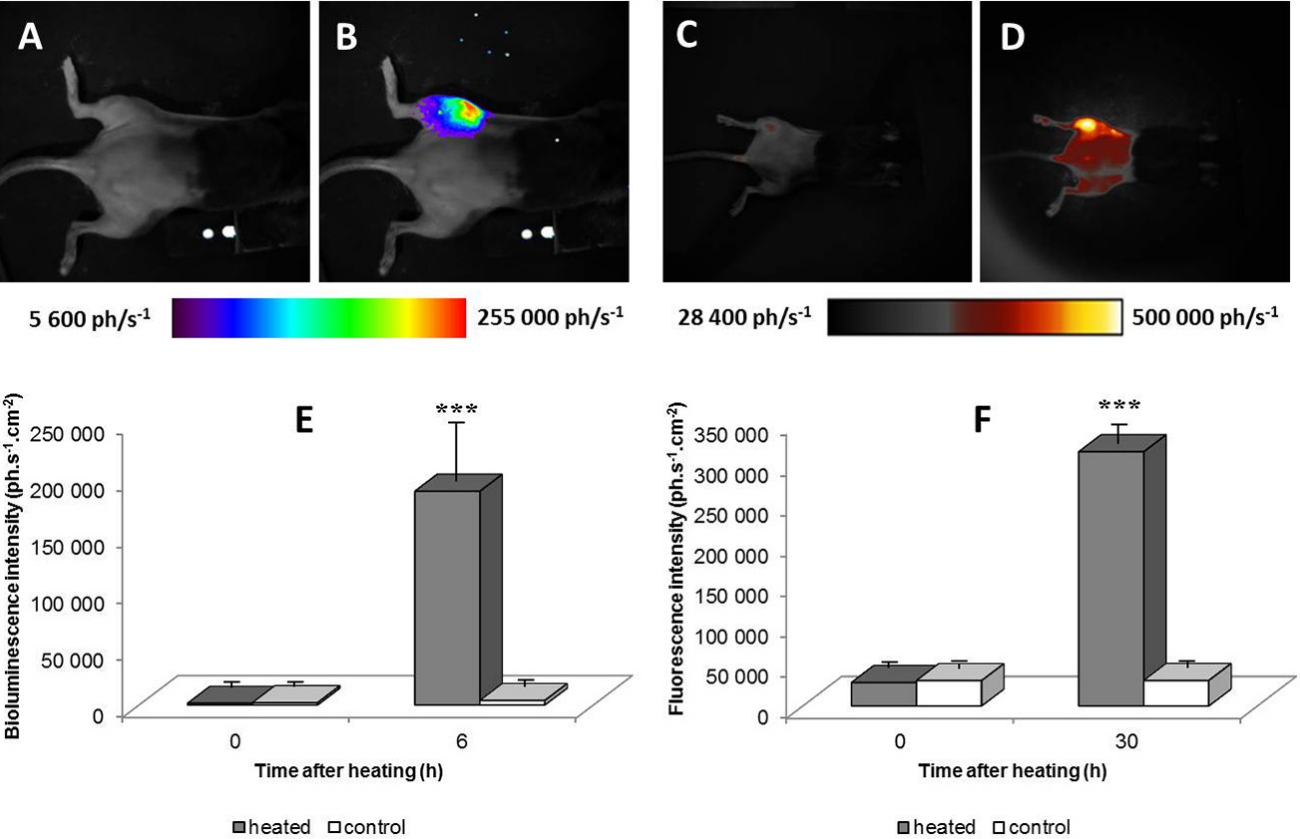
Bioluminescence images of a representative chimera mouse engrafted with bone marrow from hsp-lucF mice before A. and 6 hours after B. heating (45°C for 8 min) in water bath the left tumor-bearing leg Fluorescence images of a chimera mouse engrafted with bone marrow from hsp-lucF/hsp-mPlum mice before **C.** and 30 hours after heating **D.** The colour scales show the light intensity (photons.s^−1^.cm^−2^). Graphs represent measurement of bioluminescence **E.** (m +/− SD; *n* = 29) and fluorescent signals **F.** (m +/− SD; *n* = 5) before and after heating in heated and non-heated legs.

Chimera mice bearing tumors were assayed for heat-induced gene expression by bone marrow derived cells targeted to the tumor (*n* = 29). Mice were imaged by BLI for basal level of reporter gene expression before heat shock. As show in Fig 1A no BLI signal was detected in chimera mice and the bioluminescent signal from the left leg bearing the tumor was not different from the signal of the right leg. Six hours after heating the mouse left leg bearing the tumor, using a warm water bath (45°C, 8 min), BLI signal strongly increased in the tumor area (Fig 1B) (*n* = 29) exhibiting up to 100 fold higher signal than before heating (Fig 1E; m +/− SD; *p* < 0.01). No significant increase in BLI signal was observed after heating in C57BL6 mice bearing tumor (*n* = 3) and in chimera mice without tumor (*n* = 3).

For mice engrafted with bone marrow from hsp-lucF/hsp-mPlum mice (*n* = 5), a strong BLI signal were detected, 6 hours after heating as already reported above for hsp-lucF engrafted mice. An increase in fluorescence signal was also detected at the level of the tumor 30 hours after heating the left leg (Fig 1C & 1D) (*n* = 5). Fluorescence level was about 11 fold higher than the basal rate (Fig 1F, *p* < 0.01).

### Reporter gene expression after tumor heating using MRgHIFU

**Figure 2:**
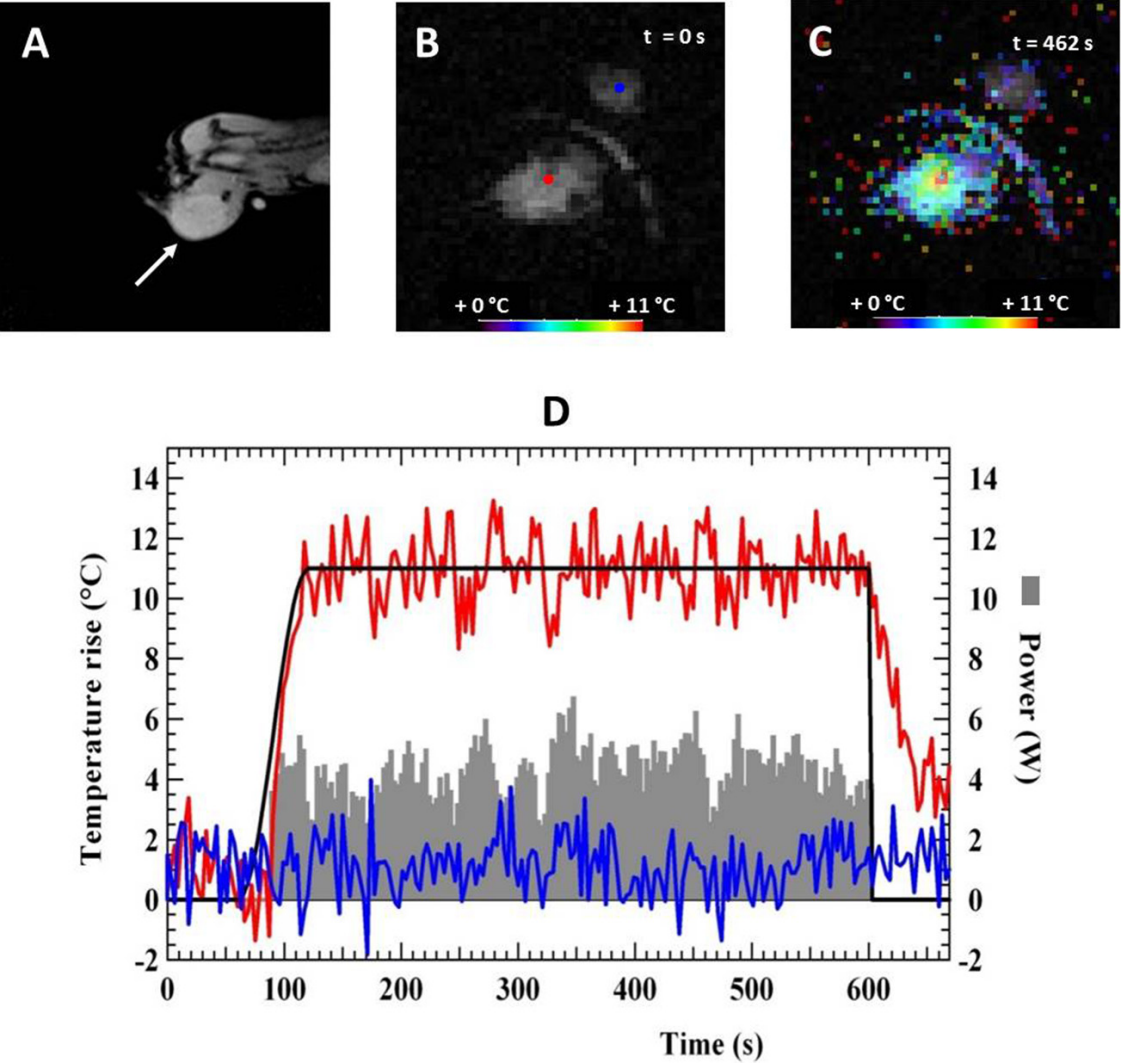
MRgHIFU heating of tumors in chimera mice showing MR T2 anatomical image A. of the tumor on the left leg of the mouse, MR temperature map before B. and at 462 s during heating C Temperature map are color-coded and superimposed on anatomical MRI image of the tumor on the left. D. Representative time course of temperature and power evolution during a heating experiment showing target temperature (black line), measured temperature at the focal point (red line), measured temperature in an agar gel next to the mouse (blue line) and applied ultrasound power during the heating experiment (grey area).

One week after water bath heating, the chimera mice engrafted with bone marrow from hsp-lucF mice and bearing tumor were assayed for local heating of the tumor using MRgHIFU (*n* = 29). The mouse was positioned on the left side with the CMT-93 tumor placed on the top of the HIFU transducer. MR images T1 and T2 (Fig 2A) were first acquired to localize the tumor and to refine animal position. Then, a sequence was run to acquire an initial set of temperature map in the tumor (Fig 2B) and to determine ultrasound focal point position using a short duration (10 s) ultrasound pulse at low power (5 W). Next, a 10 min-automatic HIFU heating was run to obtain a temperature rise of 11°C in the tumor. Temperature maps were calculated every 3 sec (Fig 2C) and HIFU power was adjusted to fit the target temperature. Fig 2D showed an example of both HIFU power and temperature evolution at the focal point. The measured temperature followed the predefined temperature profile. Standard deviation of temperature was typically +/− 1°C. The average power of ultrasound delivered to the tumor during the heating was 7 W ± 2.5 W (*n* = 29). After heating, MR T1 and T2 images were acquired and did not revealed any morphological abnormalities induced by the heating procedure.

**Figure 3:**
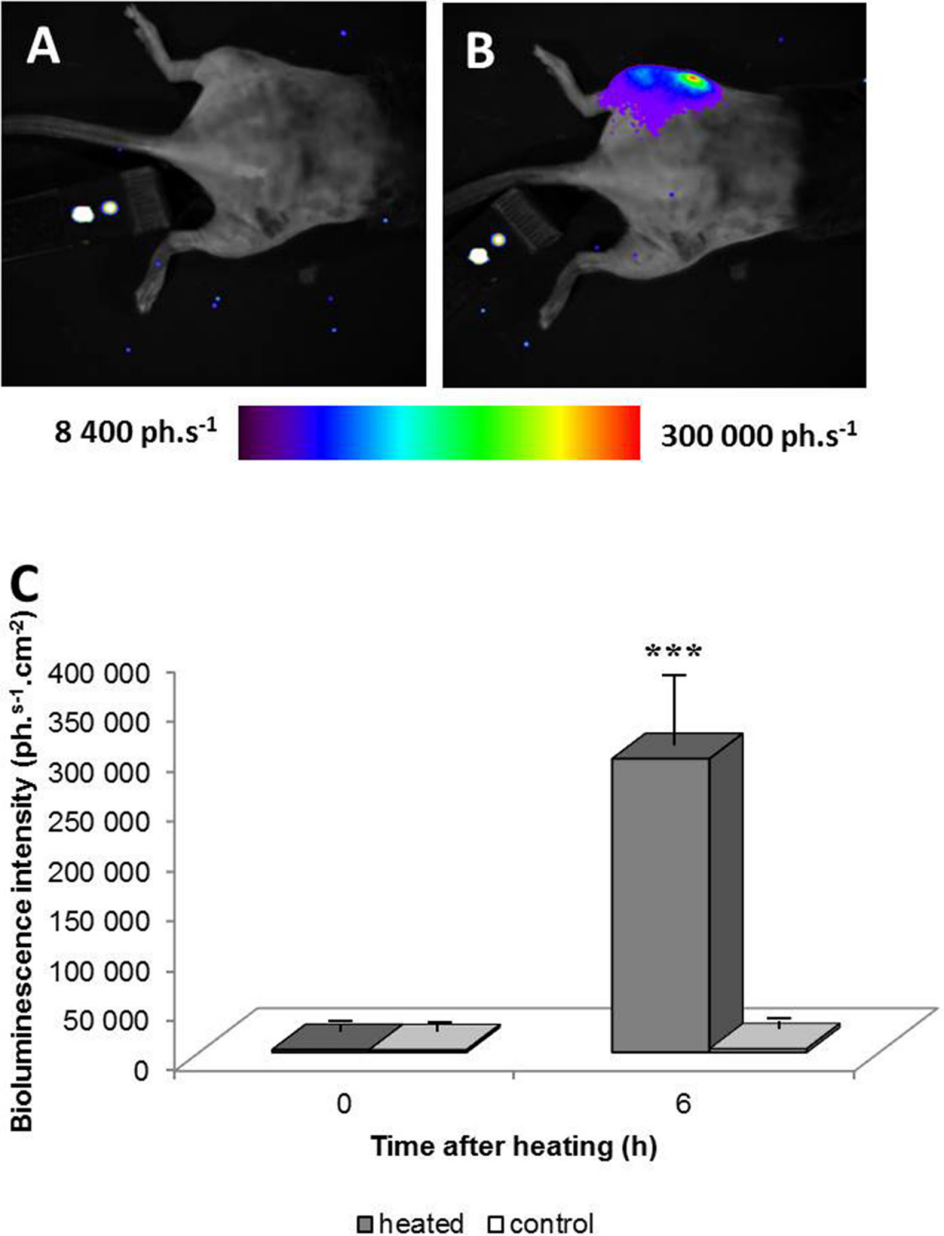
Bioluminescence images of a chimera mouse engrafted with bone marrow from hsp-lucF mice before A. and 6 hours after B. heating a tumor 45°C for 8 min using MRgHIFU Color scales represent light intensity (photons.s^−1^.cm^−2^). The graph **C.** shows measurements of bioluminescence signals before and after heating in heated and non-heated legs (m +/− SD; *n* = 29).

LucF activity before and 6 hours after tumor heating by MRgHIFU was measured by BLI. As illustrated in Fig 3, no signal was observed on the tumor before the heating procedure (Fig 3A) but a strong BLI signal was detected after heating (Fig 3B) in the tumor area. Quantification of the BLI signal 6 hours after heating revealed a highly significant increase in lucF expression in the heated tumor-bearing leg, in comparison to BLI signal measured before heating the left leg and that in the contra lateral non heated leg (Fig 3C, m +/− SD; *n* = 29, *p* < 0.01).

**Figure 4:**
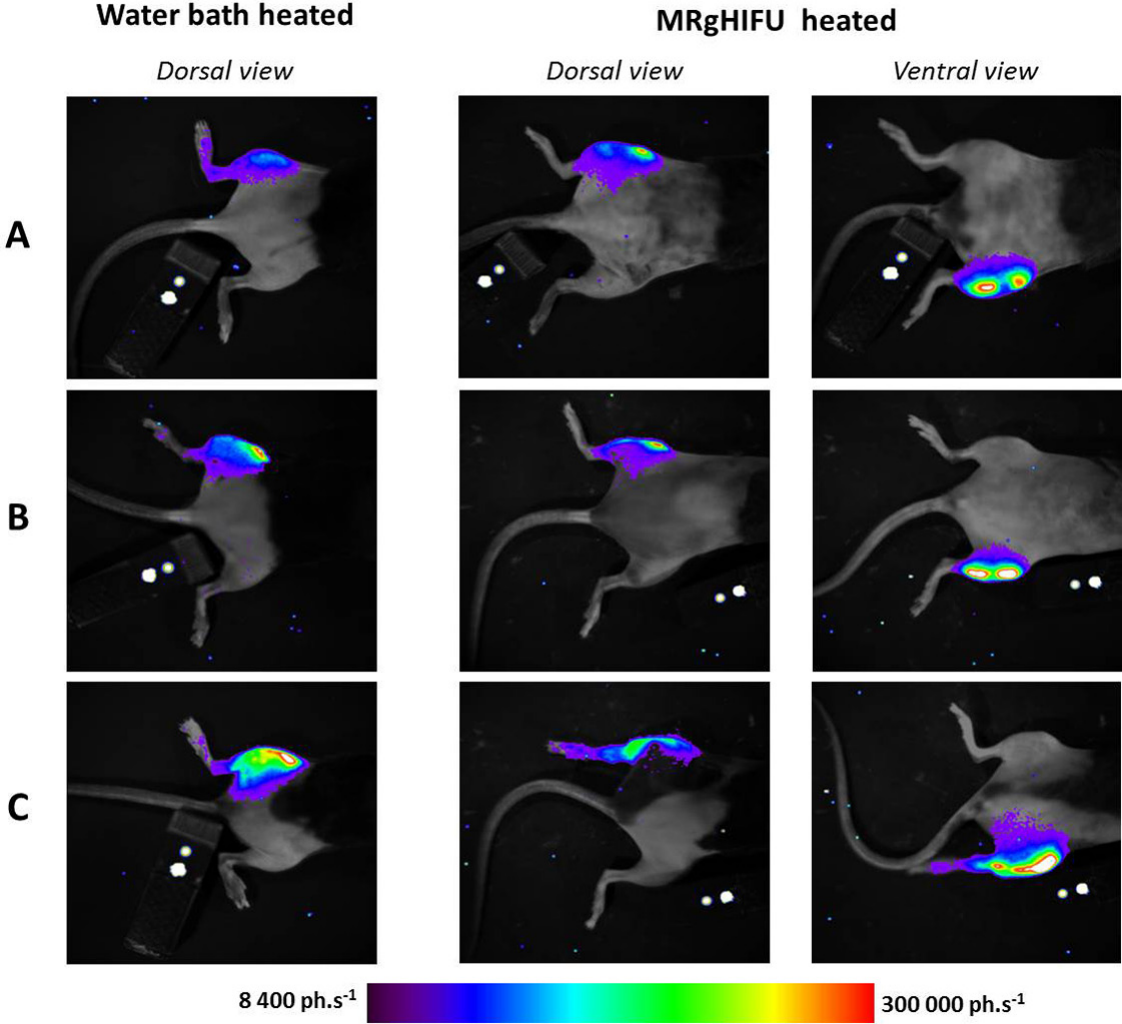
Comparison of light distribution patterns between heating methods First column shows a dorsal view after water bath heating. The second column shows dorsal view and the third column ventral view of light pattern after MRgHIFU heating performed one week later. Each row referred to the same chimera mice engrafted with bone marrow from hsp-lucF mice.

The spatial distribution of photons on bioluminescence images while heated by MRgHIFU differed from the pattern obtained after water bath heating (Fig 4). The spatial distribution of photons after MRgHIFU revealed an irregular shape and hotspots on the leg at a certain distance from the tumor region.

In mice engrafted with bone marrow from hsp-lucF/hsp-mPlum mice that were not previously heated with water bath (*n* = 5) a strong BLI signal was detected 6 hours after MRgHIFU heating of the tumor as reported above and mPlum expression was detected by FRI 30 hours after heating. Fluorescent level in the heated tumor region was (m +/− SD; 3 285 739 photons.s^−1^.cm^−2^ +/− 163 109 photons.s^−1^.cm^−2^) about 10 fold higher (*p* < 0.01) than pre-heating (325 063 photons.s^−1^.cm^−2^ +/− 17 678 photons.s^−1^.cm^−2^) and contra lateral leg fluorescence levels (293 612 photons.s^−1^.cm^−2^ +/− 10 575 photons.s^−1^.cm^−2^).

Control mice included non-depleted, non-engrafted mice with MRgHIFU heated tumor (*n* = 3), depleted, non-engrafted mice with MRgHIFU heated tumor (*n* = 3) and depleted hsp/lucF engrafted mice with tumor placed in the MRgHIFU device but without applying ultrasound (*n* = 3). No BLI signal was detected in any of these mice.

### Immunohistochemistry of reporter gene-expressing cells

**Figure 5:**
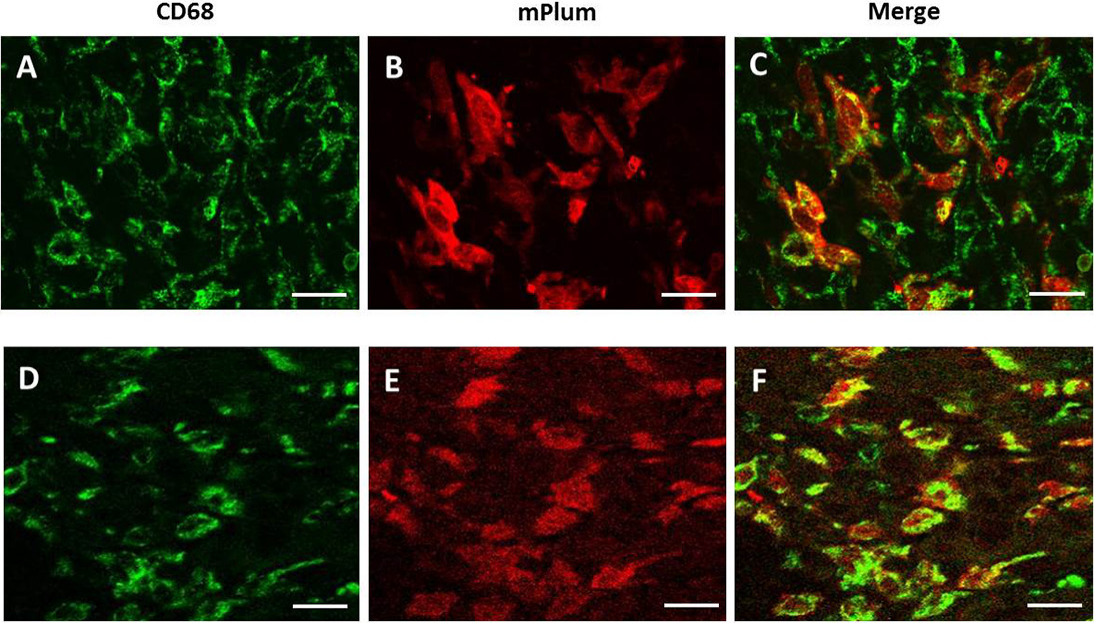
Immuno-histochemical identification of mPlum expressing cells in tumor environment Cells expressing CD68 marker (green label, **A. & D.**) and mPlum protein (red label, **B. & E.**) and merge images **(C. & F.)** 30 hours after tumor heating using water bath (A-C) or MRgHIFU (D-F). Bars represented 50 μm.

**Figure 6:**
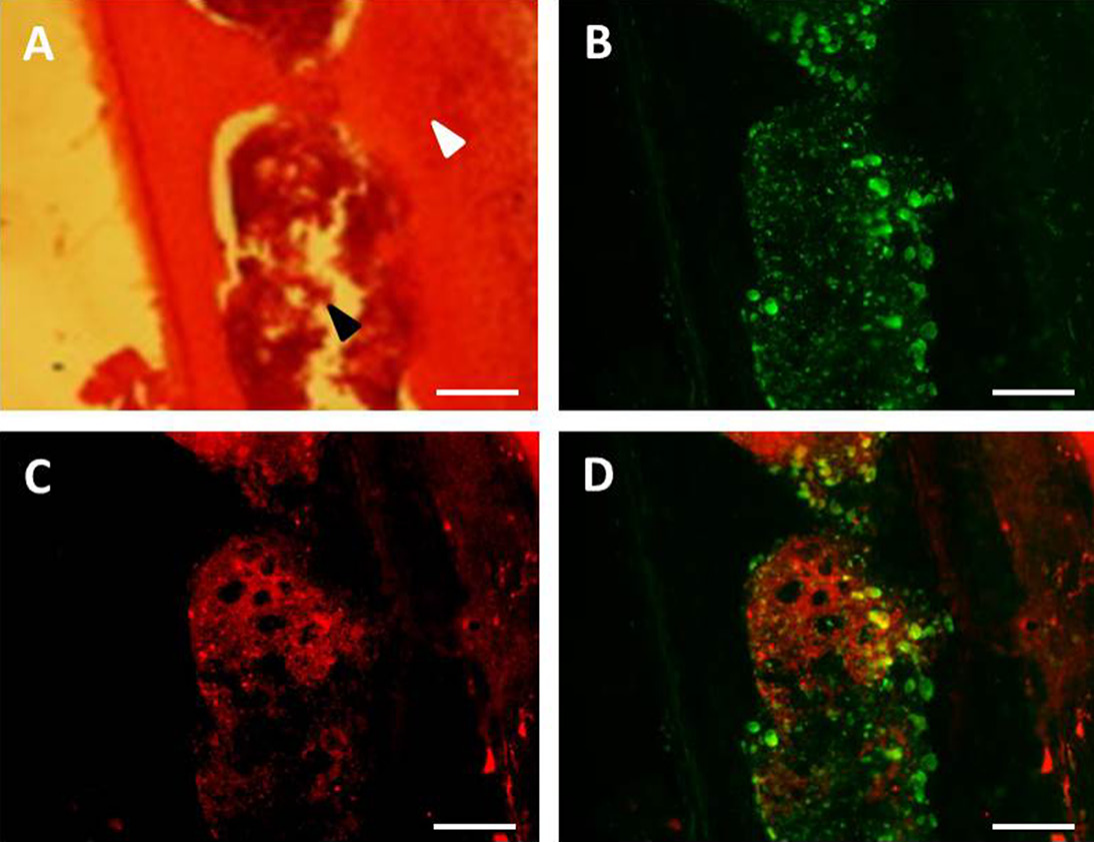
Immuno-histochemical identification of mPlum expressing bone marrow cells Cells expressing CD68 marker (green label, **B**) and mPlum protein (red label, **C**) and merge images **D.** in bone cells after MRgHIFU heating. Hematoxylin and eosin staining of the same tibia sections **A.** allowed for discriminating between bone (white arrow) and bone marrow (black arrow). Bars represented 100 μm.

Thirty hours after heating (water bath or MRgHIFU), tumors and bones from mice engrafted with bone marrow from hsp-lucF/hsp-mPlum mice, were removed and processed for mPlum and CD68 detection by immunohistochemistry. As shown in Fig 5, mPlum expressing cells were detected within the tumors after heating using either water bath (Fig 5B) or MRgHIFU (Fig 5E). CD68 expressing cells were also detected in tumor (Fig 5A & 5D). About 70% of CD68 expressing cells were also expressing mPlum (Fig 5C & 5F). The size and morphology of CD68 expressing cells in the tumors indicated they were macrophages. HES and immunohistochemistry was also performed on bone samples from MRgHIFU heated legs. As shown in Fig 6, clusters of mPlum expressing were detected in the bone marrow. CD68 expressing cells were detected in bone marrow and about 50% of them co-expressed mPlum (Fig 6D). Control mice are depleted engrafted mice with tumor and non-heated (*n* = 3).

## DISCUSSION

The tumor microenvironment is an interesting target for cancer therapies but modifying this compartment remains challenging. We show here by *in vivo* imaging, the feasibility of a gene therapy strategy that combines bone marrow-derived tumor microenvironment targeting using genetically modified bone marrow-derived cells and a spatial and a temporal control of transgene expression by non-invasive local heating.

Cells derived from hematopoietic stem cells include macrophages, dendritic cells, T and NK cells. These cells are among the major components of tumor microenvironment and therefore, constitute potential cell vectors for tumor targeted therapies. The tumor CMT-93 cells used here, like many human tumor cells [6, 9] express the chemo-attractant monocyte-1 protein (MCP-1/CCL2) [14] and many bone marrow derived cells were attracted by the tumor. Genetic modifications of the hematopoietic stem cells thus provided a way to genetically modify a large number of differentiated cells within the tumor microenvironment. Methods to modify hematopoietic stem cells *in situ* or *ex-vivo* already exist [15] and thus provided realistic translational perspectives for the strategy presented in the present work. We took advantages of a transgenic mice donor to engraft genetically-modified bone marrow cells to ensure a high level of chimerism of bone marrow derived-cells in blood and in the tumor microenvironment.

The widespread localization of circulating cells that originate from hematopoietic stem cells and their plethoric roles in healthy and diseased tissues require spatial and temporal restriction of transgene expression. It has been demonstrated that cloning a transgene under the transcriptional control of a heat-sensitive promoter combined with a local hyperthermia allows for *in vivo* control of transgene expression [12, 13]. In the current work, the transgenic mice strain used as bone marrow donor expressed 2 optical imaging reporter genes under transcriptional control of Hsp promoters. This strain has been fully characterized for its response to heat shock inducing early (peaking at 6 hours) and transient expression of firefly luciferase protein for sensitive *in vivo* detection by BLI and late (at 30 hours) and persistent mPlum fluorescent protein for *in vivo* detection by FRI and *in vitro* detection by immunohistochemistry [16]. We show here that heating activated the hsp dependent transgene expression by cells derived from the bone marrow graft that accumulated in tumor area.

MRgHIFU provided efficient non-invasive local control of hyperthermia *in vivo* in accordance with previously shown spatial and temporal control of gene expression using MRgHIFU by our group [12, 13]. Evidence of heat activation of bone marrow cells was provided by the BLI pattern showing extension of signal to the knee and the ankle. On the contrary, BLI pattern obtained in water bath heated legs was mostly centered on the tumor. Immuno-histochemical finding further confirmed Hsp activation in tibia bone marrow cells after HIFU heating but not after water bath heating. The mPlum expression was detected in CD68-positives cells which previously have been shown to constitute myeloid precursors [17]. Such a difference was attributed to a technical limitation inherent to our HIFU device, whose focal cone aperture was too large to allow selective heating of the tumor while avoiding sonication of the leg bones. Since absorption of ultrasound waves by bones is known to be higher than in muscles, substantial temperature increase of the bones can be expected [18] resulting in activation of the Hsp promoter and expression of the reporter genes by engrafted bone marrow cells [19]. In perspective of clinical application, the larger dimensions of the human targeted organs with respect to the ultrasound cone should overcome such a limitation. Alternatively, several methods have been proposed to spare bones included into a HIFU beam path while ensuring focalization into soft tissues, using either deflectors positioned on the skin [20] or selective deactivation of transducer elements to avoid illumination of the bones included into the HIFU beam path [21, 22].

The reliability and efficiency of MRgHIFU heating in tumor allowed for a temperature accuracy of about 1°C for thermo-induced gene expression. This is important as applications require both sufficient activation of the promoter and cell viability. Although a single heating condition has been used in the current work, Hsp dependent gene expression has been shown correlated with the thermal dose and thus provides a way to modulate therapeutic gene expression [23].

An important lucF signal was found in the tumor area 6 hours after heating while mPlum fluorescence was also observed in the tumor environment, but 30 hours after heating. These findings suggest that heated cells stay in the tumors area during the whole experimental procedure. Immuno-histochemical analysis identified a large number of those cells as macrophages since they expressed both mPlum and the CD68 marker. These data are consistent with the literature regarding to the abundance of tumors-associated macrophages within the tumor microenvironment [7].

With respect to the clinical relevance of the proposed method, one limitation of the current work is that engraftment occurred before the tumor implantation. However targeting of already installed tumors by bone marrow-derived cells is well documented [24]. Bone marrow engraftment is commonly used in cancer therapy. Moreover, genetic modification *in vitro* of bone marrow cells before engraftment has been also tested in a clinical trial [25, 26]. In addition, MRgHIFU technology is currently available in the clinic. In conclusion, our work provided the proof of concept for a potential therapeutic method that combined cell targeting and, spatial and temporal control of gene expression. This approach is especially relevant in the emerging context of therapeutic strategies aiming to re-educate or target tumor microenvironment [3].

## MATERIALS AND METHODS

### Animals and animals handling

Animal experiments were performed in agreement with French directives and approved by the local ethical comity (CEEA 50) under agreement A50120194. Mice were housed at the University of Bordeaux transgenic facility and maintained under 12 hours light/dark cycle with water and food *ad libitum*. Animals were anesthetized with 2% isoflurane in air (Belamont, Nicholas Piramal Limited, London, UK) and were shaved with a clipper and depilatory cream.

B6.SJL-Ptprc Pep3/BoyJ mice (Charles River laboratories, Lyon, France) were used as recipient for bone marrow engraftment and tumor implantation. The strain expressed the hematopoietic cell antigen CD45.1. Hspa1b-lucF (+/+) [27] or Hspa1b-lucF (+/+) Hspa1b-mPlum (+/−) [28] transgenic mice with C57BL/6 background were used as bone marrow donors and expressed the B cell antigen CD45.2. Transgenic mice were tested for genotype by PCR and for phenotype by heating a leg in a water bath (45°C, 8 min) then by monitoring reporter gene expression by bioluminescence and fluorescence imaging.

### Bone marrow depletion, extraction and engraftment

Sub-lethal bone marrow depletion was achieved by 2 intraperitoneal (IP) injections of busulfan (Busilvex^®^, Pierre Fabre, France) at 25 mg/Kg and 24 h intervals, 2 days before transplantation. Mice were then kept in sterile conditions to prevent any external contamination. Bone marrow cells were extracted from 6–12 week-old donor mice by flushing of tibia and femur with a syringe, filtered (70 μm) to remove tissue debris and injected intravenously (IV; 5.10^6^, 200 μL PBS) in conditioned recipient mice.

### Flow cytometer

Bone marrow engraftment was determined 1 month after engraftment on a blood drop sample using anti-CD45.1/PE (BD Pharmingen^™^, BD Biosciences, San José, California, USA) and anti-CD45.2/FITC (BD Pharmingen^™^, BD Biosciences) antibodies. Flow cytometer analysis was performed on a BD Biosciences FACScalibur™ with CellQuest software (BD Biosciences).

### Cell culture and tumor implantation

Carcinoma mouse tumor cells (CMT-93, ATCC, CCL-223) were cultured in Dulbecco's modified Eagles'medium (DMEM, Invitrogen, Carlsbad, CA, USA) supplemented with 10% fetal bovine serum (Invitrogen) and 1% antibiotic-antimycotic mixture (PSA, Invitrogen) at 37°C in 5% CO_2_. For tumor generation, 2.10^6^ cells in 100 μL PBS were injected sub-cutaneous on the left leg of grafted mice.

### Water bath heating

*In vivo* heating was performed by placing the mouse's left leg in a water bath at 45°C for 8 min. Temperature was regulated as previously described [23].

### MR thermometry and HIFU heating

Animals were positioned on the left decubitus side on a homemade platform with tumor at the focal point (1 × 1 × 5 mm^3^) of a single ultrasonic transducer element (focal length of 80 mm, external radius aperture of 120 mm; Imasonic SA, Besancon, France) immerged in a thermostated (37°C) water bath containing 0.1% (w/v) manganese. The HIFU-transducer was driven by a sinusoidal signal at 1.5 MHz (AG 1006 Series Amplifier, T & C Power Conversion, Rochester, USA). The transducer was incorporated in the bed of a 1.5 Tesla Magnetic Resonance Imaging (MRI) system (Achieva, Philips, Best, The Netherlands). A 47-mm surface receiver coil was positioned above the mouse hind leg.

Online monitoring of temperature distribution was performed with MR thermometry based on the proton resonance frequency shift technique (PRF) [29] using a multi-section fat-suppressed spectral pre-saturation with the inversion recovery rapid segmented echo planar imaging (EPI) sequence and the following parameters (EPI factor = 7, echo time = 18 ms, repetition time = 338 ms, flip angle = 30°, matrix = 63 × 64, FOV = 64 mm, 6 slices (slice thickness = 2 mm), repetition time for the stack of slices was 3 s. MRI temperature maps were calculated using the phase information in the gradient echo images and displayed [30, 31].

Potential apparent drift of the temperature (caused by drift in time of the phase of the MRI signal not related to temperature) was compensated by subtracting the average temperature in a reference region of interest (ROI) selected on a control gel outside the heated area. Short (≈10 s) low-power HIFU shots were performed to check focal point position that resulted in temperature increase in the test zone lower than 5°C.

HIFU heating experiments were performed with automatic feedback regulation of the tissue temperature at the HIFU focal point based on dynamic analysis of MRI temperature images to adjust the output power of the HIFU system (RealTI sofware, ITTech, Bordeaux, France). Heating condition consisted of a ramp time of 1 min followed by a plateau at 44°C for 8 min. The temperature increase required to reach 44°C was determined for each animal based on measurement of the rectal temperature using a MRI compatible thermometer (Luxtron, Santa Clara, CA, USA). The electric power delivered to the HIFU transducer was registered for each protocol.

### Anatomical MRI sequences

T1 and T2 weighted images were obtained before (baseline) and after (control) HIFU heating session in both sagittal and coronal orientations. The anatomical T1-weighted sequence was a Turbo Spin Echo (TSE) sequence (echo time = 80 ms, repetition time = 1200 ms, flip angle = 90°, length of echo process = 6 s, matrix = 256 × 208, FOV = 81 × 100 mm, slice thickness = 2 mm, number of accumulation *n* = 4). Anatomical T2-weighted sequence was a Fast Gradient Echo (FFE) sequence (echo time = 11.5 ms, repetition time = 450 ms, flip angle = 30°, matrix = 256 × 208, FOV = 81 × 100 mm, slice thickness = 2 mm, number of accumulation = 2).

### Optical imaging acquisition and analysis

Bioluminescence imaging (BLI) and fluorescence reflectance imaging (FRI) were performed using a NightOWL II LB 983 system equipped with a NC 100 CCD deep-cooled camera (Berthold Technologies, Bad Wildbad, Germany). For BLI, mice were injected intra-peritoneally with D-luciferin (2.9 mg in 100 μL sterile PBS, Promega), sedated at 5 min post injection and imaged at 8 min. Bioluminescence images (2 min integration period, 4 × 4 binning) and photo (100 ms exposition) were taken in prone and supine positions, respectively. A low light emitting standard (Glowell, LUX biotechnology, UK) was placed next to the animal during each image acquisition as a quality control. For FRI, images (1 s exposition, 1 × 1 binning) and photographs (100 ms exposition) were acquired. Excitation was performed at 590/20 nm and fluorescence emission was detected at 680/30 nm. Quantification was done by placing a ROI manually on the tumor and measuring the mean light intensity (in photons.s^−1^.cm^−2^) within the ROI using Indigo software (Berthold Technologies). Pseudo-color images were generated using ImageJ software.

### Immunohistochemistry

Mice were euthanized and fixed by intracardiac perfusion with 4% of paraformaldehyde (PFA, Sigma-Aldrich, St. Louis, MI, USA) solution in PBS buffer (Invitrogen). Tumors and bones were removed, post-fixed in PFA 4%, dehydrated in sucrose 30% and bone samples decalcified in EDTA 10% (Promega) then frozen and stored at −80°C. Slices of tumors (30 μm) and bones (40 μm) were obtained and immersed in a cryoprotective solution (50% PBS, 20% glycerol and 30% ethylene glycol). Hematoxylin and eosin staining was used to determine bone marrow cytology.

Slices were incubated with a rat antibody recognizing mouse CD68 (diluted 1:250, Abcam, Cambridge, UK) and a rabbit polyclonal DsRed antiserum (diluted 1:250, Living Colors, Invitrogen) at room temperature overnight. Sections were then incubated with biotinylated mouse absorbed goat anti-rat IgG (diluted 1:200, Vector Vectashield, Burlingame, California, USA) for 2 h at room temperature followed by donkey anti-rabbit IgG (diluted 1:200, Invitrogen) conjugated to Alexa546 (Invitrogen) and Streptavidin-Alexa488 (Invitrogen) for 2 h at room temperature in the dark. Sections were covered with DAPI-containing mounting medium (Vector Vectashield). Tumors were first analyzed using epifluorescence microscopy (Leica DM 5500B, Leica, Wetzlar, Germany) before being examined on a confocal microscope (Leica TCS SP2). Bones were analyzed using epifluorescence microscopy. All images were acquired with Metamorph software and processed using ImageJ software.

### Statistical analysis

Comparison of the expression levels of the two reporter gene at different time were evaluated using unpaired, heteroscedastic, one-tailed *t*-test. Statistical significance (***P < 0.01, **P < 0.05, *P < 0.1).
